# Effect of Microcapsules with Waterborne Coating as Core Material on Properties of Coating for Tilia Europaea and Comparison with Other Microcapsules

**DOI:** 10.3390/polym13183167

**Published:** 2021-09-18

**Authors:** Xiaoxing Yan, Yu Tao, Xingyu Qian

**Affiliations:** 1 Co-Innovation Center of Efficient Processing and Utilization of Forest Resources, Nanjing Forestry University, Nanjing 210037, China; 2 College of Furnishings and Industrial Design, Nanjing Forestry University, Nanjing 210037, China; taoyu@njfu.edu.cn (Y.T.); qianxingyu@njfu.edu.cn (X.Q.)

**Keywords:** waterborne coating, microcapsule, self-healing, core material, clad ratio

## Abstract

Urea formaldehyde was used as wall material and waterborne coatings as a core material to prepare microcapsules. So as to explore the influence of mass ratio of core to shell, reaction temperature and standing time on the performance of microcapsules, the orthogonal test of three factors and two levels was put into effect. The orthogonal experimental results showed the mass ratio of core to shell was the most important factor. With the increase of the mass ratio of core to shell, the output and clad ratio of microcapsules increased first and then decreased. The microcapsule with the mass ratio of core to shell of 0.67:1 had better appearance, output, and encapsulation performance. The optical properties of waterborne wood coating with the microcapsules of waterborne coating as core materials did not decrease significantly, while the hardness, impact resistance, and toughness were improved. At the same time, the microcapsules have a certain self-repairing effect on coating micro-cracks. Compared with the properties of waterborne coatings with other microcapsules, the coating with waterborne coating as core material has better comprehensive performance. The results provide a new research idea for the performance enhancement and self-healing of wood waterborne coating.

## 1. Introduction

In the process of using the coating, owing to the changes of environmental factors (such as light, temperature, humidity), the low toughness, the poor adaptability, and the complex process, the micro-cracks are generated and expanded inside the coating, and even destroy the internal structure of the coating, thus influencing the operation life of the coating and wood [1,2,3]. Because of the unique wet swelling and drying shrinkage and dimensional instability of wood [4], the microcapsule self-healing technology has a broad prospect in wood materials [5,6]. When the coating [7] is cracked by the external force, the microcapsule is cracked and the core material healing agent is released [8], which can enhance the performance of wood and prolong its service life. Waterborne acrylic coating is a commonly used waterborne coating [9,10]. It uses water as the dispersion medium, and does not contain formaldehyde and heavy metals [11,12]. It is non-toxic and has no pungent smell, and its harm to human health and environmental pollution is relatively small, so it can meet the increasingly stringent environmental protection requirements [13,14,15].

At present, the microcapsules have been widely used in wood materials [16,17]. In the combination of microcapsules and wood materials, Jeong et al. [18] used microencapsulated phase change materials to increase its heat storage, and introduced them into wood-based panels by physical mixing of microcapsules and adhesives, in order to produce high thermal efficiency wood flooring. Pinkl et al. [19] further proposed that if the self-healing material is added to repair the damaged adhesive joint, the adhesive closer to the bonding line may be better. He et al. [20] used microwave expansion and other pretreatment technologies to increase the gap of wood to achieve the purpose of introducing larger diameter microcapsules. For the application of microcapsules in wood functional materials [21], it is mainly explored how to introduce microcapsules into wood or wood materials. However, this combination process is more complex. According to the requirements of wood outputs for single or composite functional characteristics, microcapsule are evenly introduced into wood and wood materials, or attached to the surface of wood and wood materials by surface modification technology [22,23,24]. In the application of microcapsules to self-healing coatings [25], Siva et al. [26] used linseed oil and mercapto benzothiazole as core materials, and compounded urea formaldehyde microcapsules by emulsion polymerization. The self-healing ability of the anticorrosive coating with microcapsules was discussed by SVET. Lang et al. [27] encapsulated linseed oil in polyurea formaldehyde (PUF) shell and prepared a self-healing coating on account of microcapsules successfully. Meanwhile, the influence of the amount of microcapsules on the repair property of the paint-coat was explored. Yang et al. [28] explored a new self-healing system based on microcapsule organogels, where there would be no damage in the healing area. The self-healing coating was prepared by microencapsulation of urea formaldehyde polymer into polymer coating. As for the application of microcapsule in self-healing coating, scholars mainly explored its role in metal surface anti-corrosion coating, and often used linseed oil and other dry oil as core material repair agent, while there are few studies on the application of microcapsules in coatings of wood surface [29]. In order to simplify the combination of wood and self-healing microcapsule, the self-healing microcapsule with waterborne coating as the core material is applied to the coating on the surface of wood in this paper. It is of great significance to apply self-healing microcapsules to prepare self-healing coatings for wood products. The core material of self-healing microcapsules has an important influence on the properties of microcapsules. If the core material repair agent and coating are two different substances, after the coating has micro cracks, when the core material repair agent is curing and healing micro cracks, the combination of the repair agent and coating may produce interfacial stress, which affects the repair effect of micro cracks. Therefore, the choice of microcapsule core material will not only affect the performance of the microcapsule itself, but also have a great impact on the self-healing performance of the coating. As the wall material of microcapsules, urea formaldehyde resin has the advantages of low cost, mature process, and good coating of core materials. However, free formaldehyde gas may be released in the preparation process, causing harm to the human body. Therefore, it is necessary to control the content of formaldehyde and reduce the release of formaldehyde.

The performance of waterborne coating is better than that of single resin because it contains resin, additive, and other compounds. Using waterborne acrylic coating as the core material, the repairing agent can be cured at room temperature to form a coating after micro cracks [30,31]. Because the composition of the core material is consistent with that of the waterborne coating itself, the interface compatibility will be better when the crack is healed, and the performance of the coating can be further improved. In order to further explore the effect of the core material, this paper used waterborne coating itself as the core material repair agent to prepare microcapsules, and its process parameters were optimized. Then, the prepared microcapsules were added to waterborne coating to explore the effect of the self-healing microcapsules on the coating properties. On this basis, the coating properties of microcapsules were compared with those of other microcapsules with different core materials reported in the literature [32,33,34]. The main objective of the research is to explore the influence of different core materials on the coating and obtain better self-healing effect, so as to provide the basis for the application of self-healing microcapsules in wood materials in the future.

## 2. Materials and Methods

### 2.1. Materials

The chemical reagent information required for the experiment is shown in Table 1. Nippon waterborne acrylic coatings, which are mainly composed of waterborne acrylic copolymer, additives, and water, was provided by Nippon (Changzhou) Co., Ltd., Changzhou, China. Tilia europaea with size of 100 × 50 × 4 and uniform color were provided by Guangdong Yihua life science and Technology Co., Ltd., Shantou, China.

### 2.2. Preparation of Microcapsules

Firstly, according to the related literature of microcapsules [35], three vital factors affecting the preparation of microcapsules were determined. As shown in Table 2, the orthogonal test of 3 factors and 2 levels was conducted. Two levels of mass ratio of core to shell, reaction temperature, and standing time were chosen to prepare urea formaldehyde-coated waterborne coating microcapsules.

**Figure 1:**
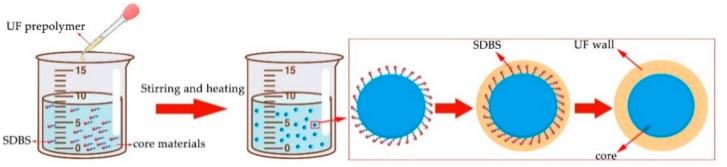
The formation process of microcapsules.

Samples 1–4# microcapsules of the orthogonal test were prepared, and the details of each substance dosage were listed in Table 3. Taking sample 1# of orthogonal experiment as an example, the preparation process of microcapsules was explained. The formation process of microcapsules is shown in Figure 1. The first step was the synthesis of urea formaldehyde (UF) prepolymer: after 20.0 g urea was poured into the beaker, 27.0 g of 37.0% formaldehyde solution was added into it and fully stirred. Then, the triethanolamine was put in slowly to regulate the pH to 8.5–9.0, and the mixture was continuously stirred at the rate of 100 rpm in a stationary temperature water bath at 70 °C for 30 min to obtain a faintly thick and transparent UF prepolymer solution. The second step was the emulsification of waterborne coatings for core material: 0.975 g of SDBS was put into distilled water and stirred thoroughly to obtain a mixture with concentration of 1.0% as emulsifier. Next the 12.5 g waterborne coating was mixed with 97.0 mL emulsifier solution. The mixture was emulsified for 60 min at the rate of 1200 rpm and 50 °C reaction temperature to gain steady emulsion. The last step was microencapsulation: the wall material prepolymer was slowly dropped into the core material emulsion obtained in the second step at the speed of 300 rpm, then citric acid solution was dropped gradually, and the mixture was continuously stirred until the citric acid crystal was completely dissolved. After adjusting the pH value to 3.0–3.5, the temperature was tardily increased to 50 °C and kept for 2.5 h. After 1 d of standing time, the production was filtered and distilled water was used at the same time to wash off the surplus emulsifier. Then, the production was laid into the drying oven and heated at 45 °C for 24 h. The powder gained was the required sample 1# in the orthogonal experiment. The preparation process of other microcapsules samples is consistent with the above.

Through the above orthogonal experiment, the most influential factor of the three factors on the performance of microcapsules and the better preparation scheme of microcapsules were determined, and next the independent test was developed for the most influential element. The experimental materials, equipment, and preparation method of microcapsules remained unchanged. The reaction temperature was regulated to 70 °C, and the standing time was set at 5 d. As an independent variable, the mass ratio of core to shell was 0.42:1, 0.50:1, 0.58:1, 0.67:1, 0.75:1, 0.83:1, and 0.92:1 (samples 5–11#), respectively. The consumption details of each substance are shown in Table 3.

### 2.3. Preparation of Coating with Microcapsules

According to the relevant literature, when the mass fraction of epoxy resin microcapsule, shellac microcapsule, and waterborne acrylic acid microcapsule in waterborne coating is 10.0%, the coating performance is better [32,33,34]. Therefore, in order to carry out the subsequent comparative analysis, the optimized mass ratio of core to shell of 0.67:1 waterborne coating microcapsules were added to the waterborne coating with a mass fraction of 0 and 10.0%, respectively, and stirred evenly. The prepared coating was applied on Tilia europaea substrate to form a uniform coating on its surface. After the coating was completely dry, 800 mesh sandpaper [36] was used to grind the paint coating to make it smooth and the surface was mopped up with dry cloth. The above procedure was performed three times in the same way to obtain a coating thickness of about 60 μm.

### 2.4. Testing and Characterization

In the aspect of micro morphology of microcapsules, a Zeiss Axio scope A1 optical microscope (OM) and a FEI Quanta−200 scanning electron microscope (SEM), produced by FEI Company, Hillsboro, OR, USA, were employed for characterization. In terms of chemical composition, A VERTEX 80 V Fourier infrared spectrum analyzer (FTIR), produced by Bruker (Beijing) Scientific Technology Co., Ltd., Beijing, China, was applied for analysis. For the microcapsule clad ratio test, the mass of microcapsule (*m*_1_) was fully ground and soaked in ethyl acetate for 72 h. The solution should be changed every 24 h. Finally, the filtered product was washed with deionized water, and the mass of the residue (*m*_2_) after drying was weighed. The encapsulation rate (c) of microcapsules was figured out by Formula (1).

(1) $$c=\frac{m_{1}-m_{2}}{m_{1}}\times 100\%$$

In the aspect of coating optical performance, the chromaticity value of waterborne coating on Tilia europaea surface was measured by RM200-PT01 color detector (Pantone Inc., Carlstadt, NJ, USA). The HG268 trigonometric glossmeter (Shenzhen Forbes Instrument Co., Ltd., Shenzhen, China) was applied to test the gloss of waterborne coating film, and the gloss of coating at 60° incidence angle was recorded. As for the mechanical performance of the coating, the pencil with known hardness label was applied to characterize the hardness of the coating. In the process, the hardness of the pencil was gradually increased until the surface of the coating appeared defects such as plastic surface or cohesive damage. The pencil hardness of the coating was expressed by the hardest hardness that does not cause a scratch of 3 mm or more. The QFH-HG600 film scribbler (Guangzhou Yuanxiao Marine Equipment Co., Ltd., Guangzhou, China) was operated to characterize the adhesion of the coating, and the resistance of the coating falling off from the substrate was evaluated when the coating penetrated the substrate with a right angle grid pattern. According to the surface appearance of the cross cutting area, it can be divided into six grades: 0, 1, 2, 3, 4, and 5, and the first three grades were satisfactory. The QCJ film impact test instrument (Tianjin World Expo Weiye chemical Glass Instrument Co., Ltd., Tianjin, China) was applied to test the coating impact resistance. A 1.0 kg heavy hammer was fixed at different heights that were less than 50 cm, and next the heavy hammer was allowed to fall naturally to impact the test piece to observe whether cracks and spalling phenomena appeared on the surface of coating. Finally, when there was no crack in the coating, the maximum height of the hammer was the impact strength of the coating. The tensile strength of the coating was tested by AG-IC100KN universal testing machine (Guangzhou Kehui Instrument Co., Ltd., Guangzhou, China). Then, the elongation at break of the coating was counted by the tensile strength. All the tests were repeated 4 times, and the error was less than 5.0%.

## 3. Results and Discussion

### 3.1. Analysis of Orthogonal Test Results

#### 3.1.1. Analysis of Micro Characteristics of Microcapsules

**Figure 2:**
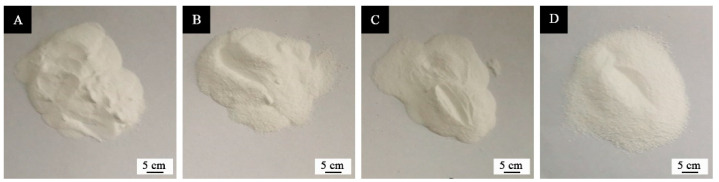
The macroscopic images of microcapsule powder: (**A**) sample 1#, (**B**) sample 2#, (**C**) sample 3#, (**D**) sample 4#.

**Figure 3:**
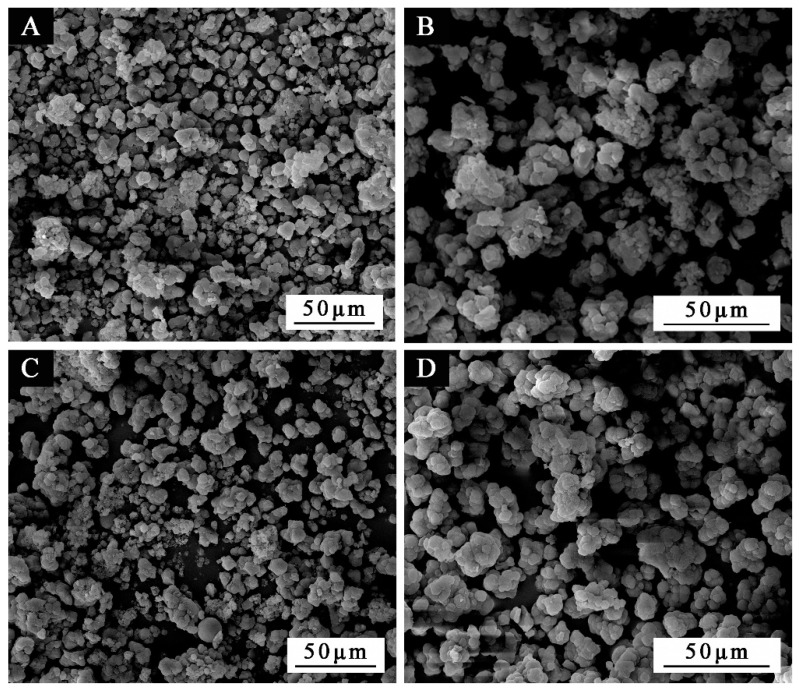
The SEM morphologies of microcapsules under different orthogonal experimental parameters: (**A**) sample 1#, (**B**) sample 2#, (**C**) sample 3#, (**D**) sample 4#.

The macroscopic images of microcapsule powder are shown in Figure 2. The SEM appearance of the orthogonal samples 1–4# in Table 3 was displayed in Figure 3. The morphology of sample 4# microcapsule (Figure 3D) is relatively good, followed by sample 1# (Figure 3A). Sample 1# has a general coating condition, with some agglomeration, but the particle size is basically uniform, about 5–8 μm. Sample 2# (Figure 3B) has a poor coating condition, with more urea formaldehyde precipitation and agglomeration, and the particle size is about 8–10 μm. Sample 3# (Figure 3C) has a general coating, with less precipitation and agglomeration, and its particle size is about 5–8 μm. Sample 4# microcapsule is coated well with a little agglomeration, and the particle size is about 8 μm. It can be found that the UF resin coated waterborne coating microcapsules whose particle size was approximately 8 μm have been successfully prepared.

#### 3.1.2. Infrared Analysis of Components in Microcapsules

**Figure 4:**
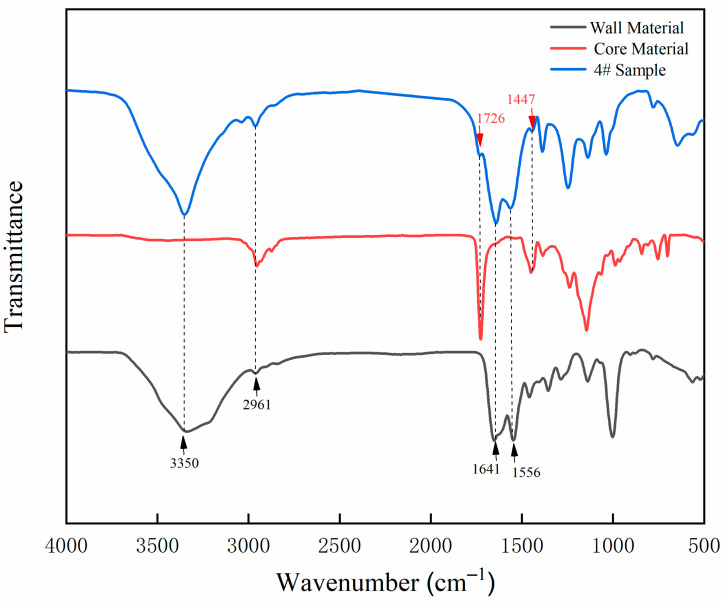
The FTIR spectrogram of wall material, core material, and sample 4# microcapsule.

Figure 4 shows the FTIR spectrogram of wall material, core material and orthogonal test sample 4#. The special absorption peaks at 3350 and 1556 cm^−1^ are assigned to N-H and C-N functional groups, which belong to the functional groups of UF resin. The absorption at 1641 cm^−1^ is consistent with the stretching vibration of C=O functional group in UF resin. The absorptions at 2961 and 1447 cm^−1^ are the special absorption peaks of C-H. The special absorption at 1726 cm^−1^ belongs to the characteristic absorption of C=O functional group in the waterborne acrylic, and the corresponding peak also appears in the infrared spectrum of sample 4# in the orthogonal test. It is confirmed that the corresponding UF resin and waterborne acrylic exist in the prepared microcapsule, and the composition is not damaged. It proves the successful preparation of UF resin cladding waterborne coating microcapsules.

#### 3.1.3. Output Analysis of Microcapsules

The microcapsule samples 1–4# made by orthogonal experiment were weighed, and the range (R) and variance results of output are shown in Table 4 and Table 5. When the mass ratio of core to wall and the standing time increased, the output of the microcapsule increased, and when the reaction temperature increased, the output of the microcapsule decreased. It can be found from the Table 4 that sample 3# microcapsule has the largest output at 33.32 g. From the range results, it can be seen that in the three factors of the mass ratio of core to shell, reaction temperature, and standing time, the mass ratio of core to shell makes the greatest influence on the output of urea formaldehyde-coated waterborne coating microcapsules, followed by the standing time. Only from the results of output, the better preparation parameters of the microcapsule are mass ratio of core to shell of 0.67:1, the reaction temperature of 50 °C and the standing time of 5 d. In Table 5, F ratio was obtained by comparing the square sum of the average deviation of each factor with the square sum of the average deviation of the error. This ratio reflected the influence of each factor on the test results. If F ratio was greater than the F critical value, it showed that this factor had a significant influence on the test results. It can be seen from Table 5 that the F ratio of the mass ratio of core to shell is greater than the F critical value, indicating that the mass ratio of core to shell has a significant impact on the output of microcapsules. 

#### 3.1.4. Clad Ratio Analysis of Microcapsules

The range and variance of clad ratio of UF resin cladding waterborne coating microcapsules made by orthogonal test are displayed in Table 6 and Table 7. When the mass ratio of core to wall, the reaction temperature, and the standing time increased, the clad ratio of the microcapsule increased. Sample 3# of the microcapsule has the highest clad ratio of 43.0%. On the basis of the variance results of the clad ratio, it is obvious that the mass ratio of core to shell has the greatest influence on the clad ratio of the microcapsules, followed by the standing time. Based on the results of clad ratio, it can be found that the mass ratio of core to shell of 0.67:1, the reaction temperature of 70 °C and the standing time of 5 d are the optimum preparation process of the microcapsules.

The orthogonal test results for this kind of microcapsule were comprehensively analyzed. Among the three elements, the mass ratio of core to shell is the most important element affecting the output and clad ratio results of microcapsule. In order to further optimize the appearance and optical and mechanical properties of microcapsules, a single factor test was carried out for the most significant influencing factor (mass ratio of core to shell) according to the above orthogonal experimental results. Based on the analysis of output results, it can be determined that the best levels of the two other factors are reaction temperature of 50 °C and standing time of 5 d, respectively, and the best levels of the analysis of clad ratio results are 70 °C and 5 d. Compared with the output, the clad ratio is a more important factor in judging the performance of microcapsules. Because the micro morphology of sample 4# microcapsule was the best, the reaction temperature was regulated to 70 °C in the process of preparing sample 4#, finally the reaction temperature was adjusted to 70 °C and the standing time was set at 5 d in the single factor experiment.

### 3.2. Analysis of Single Factor Test Results

#### 3.2.1. Analysis of Micro Characteristics of Microcapsules

**Figure 5:**
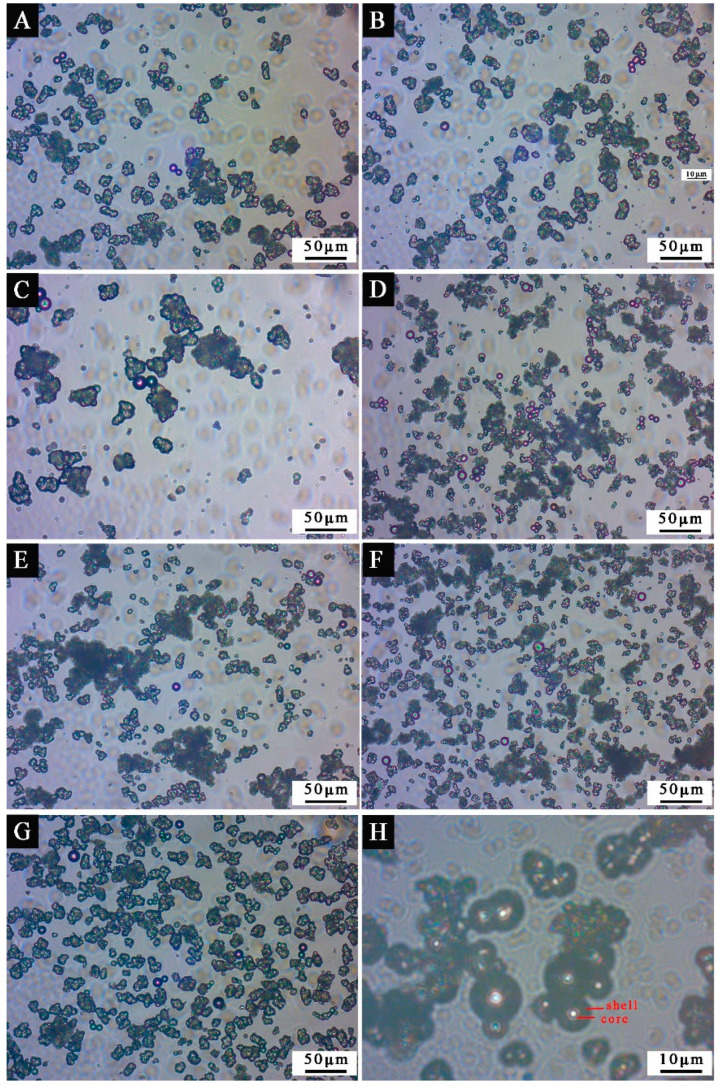
The OM appearance of microcapsules by single factor experiment: mass ratio of core to shell (**A**) 0.42:1, (**B**) 0.50:1, (**C**) 0.58:1, (**D**) 0.67:1, (**E**) 0.75:1, (**F**) 0.83:1, (**G**) 0.92:1, (**H**) 0.67:1.

**Figure 6:**
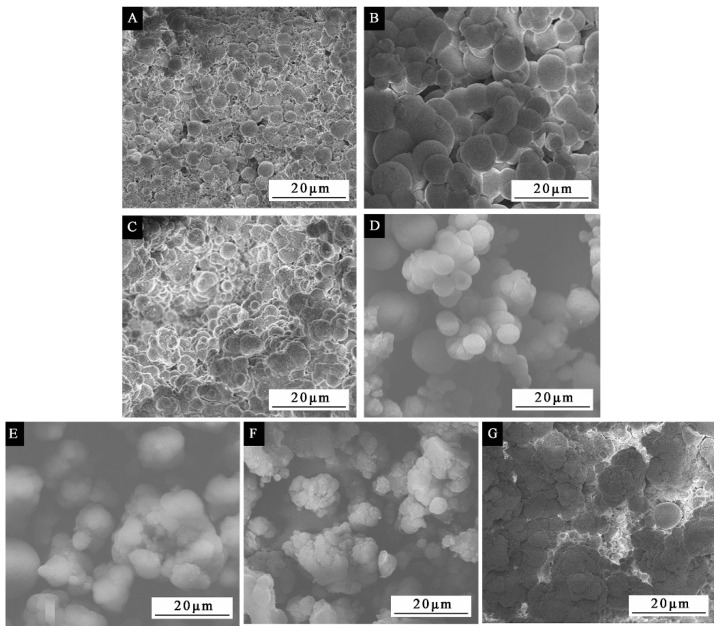
The SEM appearance of microcapsules by single factor experiment: mass ratio of core to shell (**A**) 0.42:1, (**B**) 0.50:1, (**C**) 0.58:1, (**D**) 0.67:1, (**E**) 0.75:1, (**F**) 0.83:1, (**G**) 0.92:1.

The OM and SEM of microcapsules optimized by single factor experiment with mass ratio of core to shell are displayed in Figure 5 and Figure 6. Figure 5H is the magnification image of Figure 5D. It can be seen from Figure 5H that the outside of the microcapsule sphere is the dark part, which is the wall material, and the bright spot of the microcapsule is the core material. The figure shows that the microcapsules with small mass ratio of core to shell of 0.42:1, 0.50:1, 0.58:1, and 0.67:1 (Figure 6A–D) have great morphology, consistent particle size, and almost no precipitation. However, when the mass ratio of core to shell increases, the content of core material increases, and the microcapsules with mass ratio of core to shells of 0.75:1, 0.83:1, and 0.92:1 (Figure 6E–G) can be found to have serious agglomeration and precipitation. The microcapsule with mass ratio of core to shell of 0.67:1 (Figure 5D and Figure 6D) has the best micro morphology, and the particle size is 5 μm.

#### 3.2.2. Infrared Analysis of Components in Microcapsules

**Figure 7:**
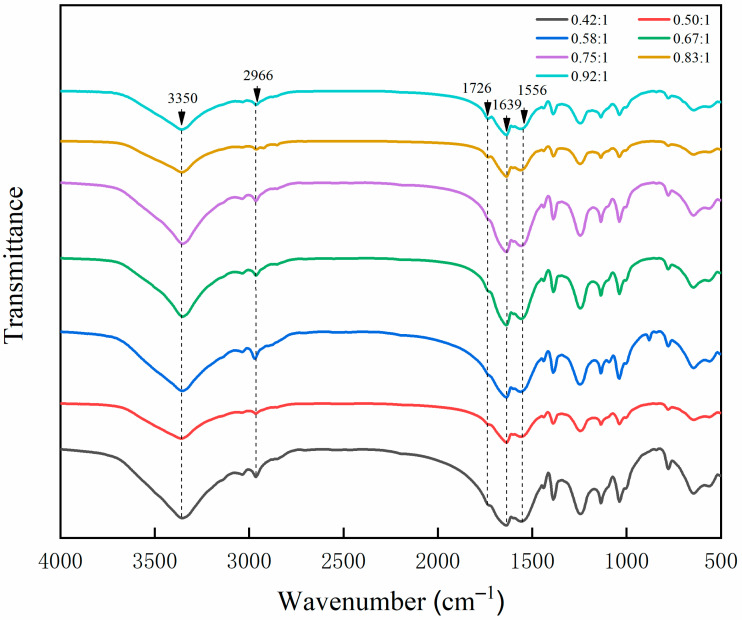
FTIR spectrum of microcapsules by single factor experiment.

Figure 7 shows the FTIR of seven kinds of microcapsules prepared by single factor experiment. The special absorption peaks of N-H and C-N functional groups are at 3350 and 1556 cm^−1^. The absorption at 1639 cm^−1^ is the stretching vibration of C=O functional group in UF resin. The absorption at 2966 cm^−1^ is the special absorption peak of C-H functional group, and the peak at 1726 cm^−1^ represents the special absorption of C=O functional group in waterborne acrylic acid coatings. It can be found from the picture that the special absorption peaks of microcapsules by different ratios of core to wall are similar, which can prove that the chemical component part of microcapsules by different ratios of core to wall is still present, and the microcapsules are successfully prepared.

#### 3.2.3. Output Analysis of Microcapsules

The output of microcapsules with seven ratios of core to shell of waterborne coatings as core material is displayed in Table 8. The output results displayed that the microcapsule with mass ratio of core to shell of 0.67:1 had the highest output. As can be seen from the table, the overall output of microcapsules basically increased first and then decreased, with the increasing of mass ratio of core to shell. When the mass ratio of core to shell increased from 0.42:1 to 0.67:1, the output of microcapsules increased from 28.93 g to 37.80 g. The reason may be that the increase of the weight of the core material can increase the weight of the wall material to cover the repair agent, thus increasing the output of the microcapsule. However, as the mass ratio of core to shell continued to increase, the output decreased. The reason may be that the increase of core material led to the increase of agglomeration and precipitation. As a result, the excess core material cannot be completely covered and the mass loss occurred during the filtration process.

#### 3.2.4. Clad Ratio Analysis of Microcapsules

The results of clad ratio of microcapsules with seven different mass ratios of core to shell in single factor experiment are shown in Table 9. From the results in the Table 9, when the mass ratio of core to shell was 0.67:1, the clad ratio of microcapsules was the highest. With the enlargement in the mass ratio of core to shell (the raise of the mass of core material), the clad ratio of waterborne coating microcapsules basically rose first then fell. When the mass ratio of core to shell increased from 0.42:1 to 0.67:1, the clad ratio of microcapsules rose from 37.0 to 49.0%, but when the mass ratio of core to shell rose to 0.92:1, the clad ratio of microcapsules fell to 32.0%. Owing to the raise of the core material mass, the content of the core material cladded in the microcapsule wall increased, which enhanced the clad ratio to a certain degree. Nevertheless, when the mass ratio of core to shell rose, the microcapsule would produce too much precipitation and agglomeration, which affected the performance of the microcapsule, resulting in the decrease of the clad ratio.

Comprehensive analysis of the results in Figure 6, Table 8 and Table 9 showed that when the mass ratio of core to shell was 0.67:1, the microcapsule had better morphology, the highest output, and the highest clad ratio, and the preparation was more successful.

### 3.3. Effect of Standing Time on the Morphology of Microcapsules

**Figure 8:**
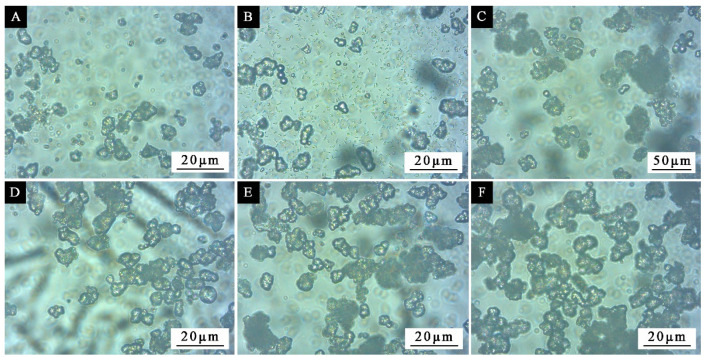
OM morphologies of microcapsules prepared under different depositing time: (**A**) 0, (**B**) 1 d, (**C**) 2 d, (**D**) 3 d, (**E**) 4 d, (**F**) 5 d.

**Figure 9:**
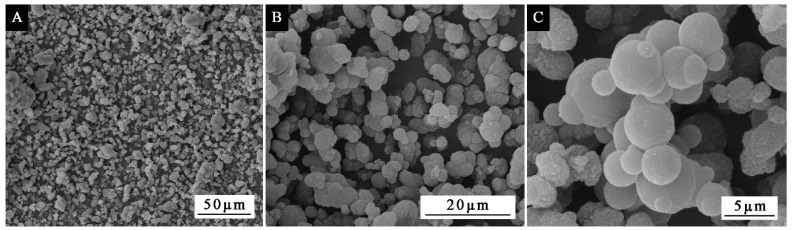
SEM morphology of microcapsule prepared under two months of deposition: (**A**) low magnification, (**B**) middle magnification, (**C**) large magnification.

In order to understand the formation mechanism of microcapsules and explore the effect of standing time on the morphology of microcapsules, the microcapsules with mass ratio of core to shell of 0.67:1 were aged for 0, 1, 2, 3, 4, and 5 d respectively. The OM of the microcapsules prepared under different standing times is shown in Figure 8. With the increase of standing time, the observed spherical particles gradually increased, and with the extension of time, the particles formed a partial agglomeration phenomenon. In order to explore whether the agglomeration phenomenon of microcapsules would seriously increase with the infinite extension of time, the prepared microcapsules were aged for two months. The SEM under different multiples is shown in Figure 9. The microcapsules had good morphology and a small amount of agglomeration and bonding. The result showed that although a small amount of agglomeration occurred with the increase of standing time, it was not serious due to the long standing time. It also showed that the standing time had little effect, and the core wall ratio was a more important factor affecting the morphology of microcapsules.

### 3.4. Influence of Microcapsules on the Performances of Waterborne Coatings

The performance of the coating is shown in Table 10. Properties of coating with 10.0% mass fraction microcapsules were compared with that of the coating without microcapsule. In terms of optical properties, the color difference of the coating increased from 0.6 to 2.0 after adding 10.0% waterborne coating microcapsules, and the gloss of the coating fell from 29.4 to 11.9% at 60° incident angle. It may be due to the uneven surface of the coating and the enhancement of diffuse reflection after adding microcapsule particles, and the influence of the color of the microcapsule itself. In terms of mechanical properties, the hardness of the coating increased from HB to 3H, the adhesion remained unchanged, and the impact resistance raised significantly from 6.0 to 13.0 kg·cm. After comprehensive analysis, it can be concluded that the performance of the waterborne coating added with 10.0% waterborne coating microcapsules did not decrease significantly, but its hardness and impact resistance were enhanced. At the same time, the elongation at break of the coating had risen from 9.94 to 16.18%, which was a significant increase. It is obvious that the toughness of the coating with microcapsule concentration of 10.0% is improved, and the existence of microcapsule can prevent the appearance of micro-cracks to a certain extent.

**Figure 10:**
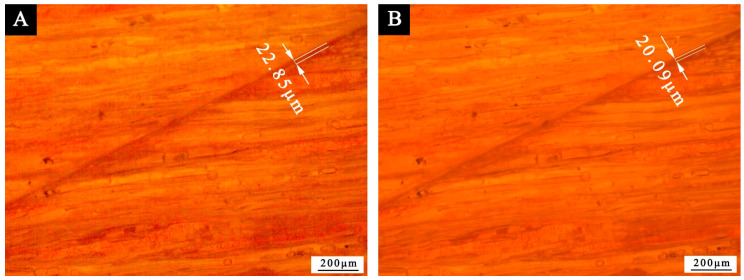
The OM diagram of paint coating without microcapsule: (**A**) 0 d, (**B**) 5 d.

**Figure 11:**
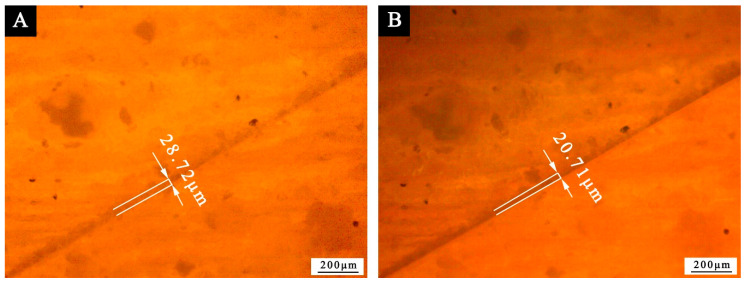
The OM diagram of paint coating with 10.0% microcapsule: (**A**) 0 d, (**B**) 5 d.

**Figure 12:**
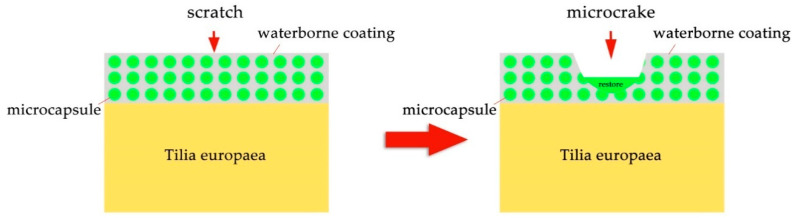
The self-repairing mechanism of waterborne coating microcapsules.

In terms of self-healing performance, the blade was used to mark the cracks on the paint coating on the surface of Tilia europaea, and the OM was used to record the cracks. After an interval of 5 d, the OM was used to observe the self-healing effect of the paint coating again. According to Figure 10, the scratch width of the coating without microcapsules changed from 22.85 to 20.09 μm, and there was no obvious change. However, it can be seen from Figure 11 that the scratch width of the coating with 10.0% microcapsules decreased significantly from 28.72 to 20.71 μm after 5 d. It can be seen that the self-healing effect of the coating with microcapsule is better than that without microcapsule. Therefore, 10.0% microcapsules have a certain self-repairing effect on the micro cracks on the surface coating of wood. The self-healing mechanism of Tilia europaea surface coating containing microcapsules is shown in Figure 12. When the coating cracks, the microcapsules in the coating will break, and the waterborne coating core repair agent in the microcapsules will flow out. The waterborne paint repair agent can be cured into a film at room temperature. The curing of waterborne coating mainly has three levels of mechanisms. The first is the volatilization of water and other film-forming aids to fully reflect the basic properties of the thermoplastic resin itself, the second is the aggregation and fusion of the emulsion particles, and the third is mutual diffusion of particles. When the temperature is higher than the glass transition temperature, the final film is formed. The micro-cracks on the coating surface can achieve a certain self-repairing effect through the curing of the core material repairing agent.

The performances of the waterborne coatings with the same content, the same wall material and different core materials reported in the literature were compared and analyzed [32,33,34]. In Table 10, the optimal mass ratio of core to shell with waterborne coating microcapsules was 0.67:1, that of waterborne acrylic acid microcapsules was 0.58:1, that of epoxy resin microcapsules was 0.83:1, and that of shellac microcapsules was 0.75:1. The results showed that the optimal core-wall ratio may be different for different kinds of core materials. From Table 10, it can be found that the optical and mechanical properties of waterborne coatings were significantly different after adding microcapsules with different core materials. Among them, the coating with waterborne coating microcapsules had the highest gloss, the coating with epoxy resin microcapsules had a higher elongation at break, and the coating with shellac microcapsule had the lowest hardness. It may be that epoxy resin is more flexible than waterborne coatings and shellac, so the elongation at break is higher. But matched with epoxy resin, the waterborne coating and shellac can be solidified at normal atmospheric temperature without heat, which is more practical for surface coating of wood furniture and wood products. However, the shellac is insoluble in water and soluble in organic solvents, which has certain difficulties in the preparation process. The coating with waterborne coating as the core material has better comprehensive properties.

## 4. Conclusions

Through orthogonal test of three factors and two levels, UF resin cladding waterborne coating microcapsules were prepared. The best reaction temperature is 70 °C and the best standing time is 5 d. The microcapsule with a mass ratio of core to shell of 0.67:1 has better comprehensive properties such as morphology, output, and clad ratio. Under the optimum preparation conditions, the clad ratio was 49.0%. Compared with the coating without microcapsules, the hardness of the coating with 10.0% mass fraction microcapsules increased to 3H, the impact strength increased to 13 kg·cm, and the elongation at break increased to 16.2%. Moreover, the microcapsules have a certain self-healing effect on micro cracks in wood surface coating. At the same time, compared with the coating with other different core materials, the coating prepared by adding microcapsules with waterborne coating as core material has better comprehensive properties. This may lay a technical foundation for the industrial application of waterborne coating microcapsules on wood surface. The future research direction is to prepare microcapsules with higher coverage rate and better repair effect for waterborne coatings on wood surface, at the same time, the wall materials will be environmental protection materials without formaldehyde emission.

## Figures and Tables

**Table 1:** Chemical reagent information in experiment.

Experimental Materials	Molecular Mass (g/moL)	CAS	Manufacturer
citric acid monohydrate	210.14	5949-29-1	Nanjing Chemical Reagent Co., Ltd., Nanjing, China
triethanolamine	149.19	102-71-6	Guangzhou Jiangshun Chemical Technology Co., Ltd., Guangzhou, China
37.0% formaldehyde	30.03	50-00-0	Guangzhou Jiangshun Chemical Technology Co., Ltd., Guangzhou, China
urea	60.06	57-13-6	Nanjing Chemical Reagent Co., Ltd., Nanjing, China
sodium dodecyl benzene sulfonate (SDBS)	348.48	25155-30-0	Xi’an Tianmao Chemical Co., Ltd., Xi’an, China
ethyl acetate	88.11	141-78-6	Tianjin Fuyu Fine Chemical Co., Ltd., Tianjin, China
anhydrous ethanol	46.07	64-17-5	Tianjin Fuyu Fine Chemical Co., Ltd., Tianjin, China

**Table 2:** Influencing factors and levels.

Level	Mass Ratio of Core to Shell	Reaction Temperature (°C)	Standing Time (d)
1	0.42:1	50	1
2	0.67:1	70	5

**Table 3:** Detailed list of various substances used in orthogonal and single factor tests.

Sample	37.0% Formaldehyde (g)	Urea (g)	Waterborne Coatings (g)	Sodium Dodecyl Benzene Sulfonate (g)	Emulsifier (mL)
1#	27.00	20.00	12.50	0.97	97.00
2#	27.00	20.00	12.50	0.97	97.00
3#	27.00	20.00	20.00	1.56	156.00
4#	27.00	20.00	20.00	1.56	156.00
5#	27.00	20.00	12.50	0.97	97.00
6#	27.00	20.00	15.00	1.17	117.00
7#	27.00	20.00	17.50	1.37	137.00
8#	27.00	20.00	20.00	1.56	156.00
9#	27.00	20.00	22.50	1.76	176.00
10#	27.00	20.00	25.00	1.95	195.00
11#	27.00	20.00	27.50	2.15	215.00

**Table 4:** Range results of microcapsule output by the orthogonal test.

Sample	Mass Ratio of Core to Shell	Reaction Temperature (°C)	Standing Time (d)	Output (g)
1#	0.42:1	50	1	28.76 ± 0.95
2#	0.42:1	70	5	29.34 ± 1.02
3#	0.67:1	50	5	33.32 ± 0.84
4#	0.67:1	70	1	32.19 ± 0.92
Mean 1	29.05 ± 1.38	31.04 ± 0.97	30.47 ± 0.78	
Mean 2	32.75 ± 0.85	30.76 ± 0.99	31.33 ± 0.83	
R	3.70 ± 0.10	0.27 ± 0.01	0.85 ± 0.01	

**Table 5:** Variance results of microcapsule output by the orthogonal test.

Factor	Sum of Squared Deviations	Degrees of Freedom	F Ratio ^1^	F Critical Value ^2^	Significance
Mass ratio of core to shell	13.727	1	180.618	161.000	*
Reaction temperature (°C)	0.076	1	1.000	161.000	
Standing time (d)	0.731	1	9.618	161.000	
Error	0.08	1			

^1^ F ratio is obtained by comparing the square sum of the average deviation of each factor with the square sum of the average deviation of the error. ^2^ F critical value is the critical value of given significant level.

**Table 6:** Range results of microcapsule clad ratio by the orthogonal test.

Sample	Mass Ratio of Core to Shell	Reaction Temperature (°C)	Standing Time (d)	Clad Ratio (%)
1#	0.42:1	50	1	33.0 ± 1.0
2#	0.42:1	70	5	37.0 ± 1.0
3#	0.67:1	50	5	43.0 ± 1.4
4#	0.67:1	70	1	40.0 ± 0.8
Mean 1	35.00 ± 1.01	38.00 ± 1.10	36.50 ± 0.90	
Mean 2	41.50 ± 0.76	38.50 ± 1.00	40.00 ± 1.18	
R	6.50 ± 0.18	0.50 ± 0.02	3.50 ± 0.06	

**Table 7:** Variance results of microcapsule clad ratio by the orthogonal test.

Factor	Sum of Squared Deviations	Degrees of Freedom	F Ratio	F Critical Value	Significance
Mass ratio of core to shell	42.25	1	160.00	161.00	
Reaction temperature (°C)	0.25	1	1.00	161.00	
Standing time (d)	12.25	1	49.00	161.00	
Error	0.25	1			

**Table 8:** Output results of microcapsules by the single factor test.

Sample	Mass Ratio of Core to Shell	Core Material Mass (g)	Output (g)
5#	0.42:1	12.5	28.93 ± 0.40
6#	0.50:1	15.0	31.22 ± 1.19
7#	0.58:1	17.5	34.35 ± 1.04
8#	0.67:1	20.0	37.80 ± 0.88
9#	0.75:1	22.5	35.57 ± 0.76
10#	0.83:1	25.0	33.43 ± 0.69
11#	0.92:1	27.5	33.47 ± 0.76

**Table 9:** Clad ratio results of microcapsules by the single factor test.

Sample	Mass Ratio of Core to Shell	Core Material Mass (g)	Clad Ratio (%)
5#	0.42:1	12.5	37.0 ± 1.2
6#	0.50:1	15.0	46.0 ± 1.4
7#	0.58:1	17.5	45.0 ± 1.3
8#	0.67:1	20.0	49.0 ± 1.8
9#	0.75:1	22.5	42.0 ± 1.2
10#	0.83:1	25.0	41.0 ± 1.3
11#	0.92:1	27.5	32.0 ± 0.6

**Table 10:** Performance comparison of waterborne coatings with different microcapsules.

Core Material	Optimum Mass Ratio of Core to Shell	Microcapsules Concentration (%)	Color Difference	Gloss (%)	Hardness	Adhesion (Grade)	Impact Resistance (kg·cm)	Elongation at Break (%)
−	−	0	0.60 ± 0.01	29.4 ± 0.8	HB ± 0	0 ± 0	6.0 ± 0.1	9.9 ± 0.2
Waterborne coating	0.67	10.0	2.00 ± 0.08	11.9 ± 0.3	3H ± 0	0 ± 0	13.0 ± 0.4	16.2 ± 0.5
Waterborne acrylic acid	0.58:1	10.0	1.80 ± 0.03	5.1 ± 0.1	2H ± 0	1 ± 0	15.0 ± 0.3	16.7 ± 0.5
Epoxy resin	0.83:1	10.0	3.50 ± 0.03	5.0 ± 0.1	5H ± 0	3 ± 0	20.0 ± 0.3	35.0 ± 1.0
Shellac ^1^	0.75:1	10.0	1.50 ± 0.04	7.8 ± 0.2	B ± 0	1 ± 0	9.0 ± 0.2	20.9 ± 0.1

^1^ Shellac is a kind of purple natural resin secreted by shellac insect after absorbing the sap of host tree.

## Data Availability

Not applicable.
